# Publicly Reported Wound Healing Rates: The Fantasy and the Reality

**DOI:** 10.1089/wound.2017.0743

**Published:** 2018-03-01

**Authors:** Caroline E. Fife, Kristen A. Eckert, Marissa J. Carter

**Affiliations:** ^1^ Department of Geriatrics, Baylor College of Medicine, Houston, Texas.; ^2^ The U.S. Wound Registry, The Woodlands, Texas.; ^3^ Strategic Solutions, Inc., Cody, Wyoming.

**Keywords:** wound healing rates, Merit-Based Incentive Payment, quality measures, real world data, qualified clinical data registry, randomized controlled trials

## Abstract

**Significance:** We compare real-world data from the U.S. Wound Registry (USWR) with randomized controlled trials and publicly reported wound outcomes and develop criteria for honest reporting of wound outcomes, a requirement of the new Quality Payment Program (QPP).

**Recent Advances:** Because no method has existed by which wounds could be stratified according to their likelihood of healing among real-world patients, practitioners have reported fantastically high healing rates. The USWR has developed several risk-stratified wound healing quality measures for diabetic foot ulcers (DFUs) and venous leg ulcers (VLUs) as part of its Qualified Clinical Data Registry (QCDR). This allows practitioners to report DFU and VLU healing rates in comparison to the likelihood of whether the wound would have healed.

**Critical Issues:** Under the new QPP, practitioners must report at least one practice-relevant outcome measure, and it must be risk adjusted so that clinicians caring for the sickest patients do not appear to have worse outcomes than their peers. The Wound Healing Index is a validated risk-stratification method that can predict whether a DFU or VLU will heal, leveling the playing field for outcome reporting and removing the need to artificially inflate healing rates. Wound care practitioners can report the USWR DFU and VLU risk-stratified outcome measure to satisfy the quality reporting requirements of the QPP.

**Future Directions:** Per the requirements of the QPP, the USWR will begin publicly reporting of risk-stratified healing rates once quality measure data have met the reporting standards of the Centers for Medicare and Medicaid Services. Some basic rules for data censoring are proposed for public reporting of healing rates, and others are needed, which should be decided by consensus among the wound care community.

**Figure:**
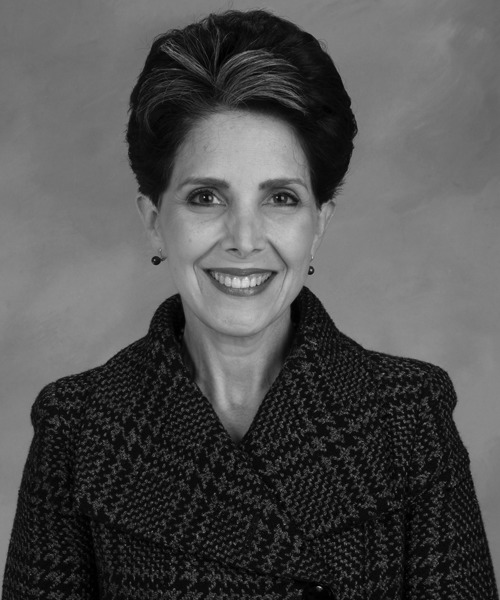
**Caroline E. Fife, MD**

## Scope and Significance

We compare real-world healing rates from the U.S. Wound Registry (USWR), a Qualified Clinical Data Registry (QCDR), with randomized controlled trials (RCTs) and publicly reported data and develop criteria for honest reporting of wound outcomes. RCT and USWR data provide convincing evidence that most wounds do not heal, whereas healing rates posted online by provider entities are so high and cover such short time frames that they appear impossible. Although some data censoring is necessary, wounds must be risk adjusted to satisfy the quality reporting requirements of the new Quality Payment Program (QPP).

## Translational Relevance

Under the Merit-Based Incentive Payment System (MIPS), practitioners must report at least one practice-relevant outcome measure to qualify for a bonus payment. Unfortunately, the Centers of Medicare and Medicaid Services (CMS) does not have wound care--relevant measures available. Although some data censoring is necessary, if quality data are reported by any QCDR, at least one risk-adjusted outcome measure must be reported to satisfy reporting requirements. As a solution to this conundrum, the USWR provides wound care–relevant quality measures, including risk-stratified healing rate measures for DFUs and venous leg ulcers (VLUs).

## Clinical Relevance

Without a standardized risk stratification method, there is considerable pressure to inflate publicly reported healing rates, because *not* to do so will make the practitioners appear less clinically capable. Based on USWR and RCT data, it is likely that in the real world, among complicated patients, healing rates better than 40.0% are not achievable. By reporting that nearly all wounds heal, we are unable to elucidate the relative contribution of specific interventions. Inflating healing rates makes it nearly impossible to develop episode-based payment models, a task upon which the future survival of the field of wound care may depend.

## Overview

### Public reporting: the fantasy of quality data

In 2016, the performance data of all practitioners participating in the Physician Quality Reporting System (PQRS) became publicly available for the first time on the Physician Compare website, although the data available lag 2 years behind the calendar year.^1^ CMS is continuing its transition to a healthcare payment system based on quality rather than quantity through the MIPS, to which the vast majority of physicians became subject on January 1, 2017.^2^ Under the MIPS, quality reporting comprises 60% of the total performance score this first year. Practitioners hoping to realize a bonus payment must successfully report six quality measures, at least one of which should be a practice-relevant outcome measure. Since there are no wound care–relevant outcome measures available from CMS, wound care practitioners find themselves like Lewis Carroll's *Alice* down the rabbit hole, wondering which way to go from here. In this “through the looking glass” conundrum, MIPS success is largely dependent on the reporting of quality measures which CMS specifically did not create for wound care providers. Happily, wound care–relevant quality measures are available through the USWR, a QCDR recognized by CMS for quality measure development and reporting under MIPS.^3,4^ By federal law, if quality data are reported by any QCDR, at least one outcome measure must be reported and must be risk adjusted [see 78 FR 43363, Section 601(b) of the American Taxpayer Relief Act of 2012].^5^ Risk adjustment is a corrective tool to “level the playing field” when reporting patient outcomes, making it possible to compare provider performance fairly.^6^ In other words, if the USWR reports “healing rates” (a logical outcome measure for wounds), it must do so using a risk stratification tool to prevent practitioners caring for the sickest patients from being penalized by appearing to have poorer outcomes than their peers. With quality performance data now public on Physician Compare,^7^ we share *Alice's* concern that words cannot simply be allowed to mean whatever anyone choses. Payers already use quality measure performance to negotiate contracted reimbursement rates, and potential employers may use quality measure data in their hiring decisions. The online platform for crowd-sourced reviews, “Yelp,” which already offers physician ratings by consumers, is negotiating with CMS to integrate PQRS performance data from Physician Compare, transforming Yelp into the driving force of consumer decision making for healthcare providers.^8^ The USWR has developed two risk-stratified healing rate measures, one for diabetic foot ulcers (DFUs) and the other for VLUs. Given the far-reaching implications of publicly reported outcome data and the federal mandates for risk adjustment of QCDR outcome measures, the time has come, as the Walrus said, “to talk of many things.” At the very least, dialogue is needed regarding the industry norms for data censoring of wound outcomes and a change in the public reporting of wound healing rates, which are currently works of fiction.

In this review, we demonstrate how data from RCTs provide convincing evidence that most patients do not heal their wounds, despite the exclusion of most serious comorbid diseases. In contrast, the healing rates posted online by various provider entities tout wound healing rates so high and over such short time frames that they can safely be classified, to paraphrase *Alice*, among the impossible things one cannot believe. The purpose of this article is to compare real-world healing rates from the USWR with RCTs and publicly reported data, and develop a reasonable strategy for honest reporting of wound outcomes, a CMS requirement under the new Quality Payment Program.

### Publicly reported quality data: believing in six impossible things before breakfast

In January 2017, we searched “wound clinic healing rates” on Google to determine publicly reported quality data by providers, hospitals, corporations, and other wound care–related businesses. The search resulted in “around 465,000 websites,” of which the first 490 were provided. None of these results comprised data from clinical trials, manufacturers of devices or drugs, or any other entity not associated with the actual delivery of care as performed by an advanced practitioner in the clinical setting. We reviewed each wound center website and included publicly reported data from the first center listed with data available from each state. To determine public data transparency, we specifically searched for and collected data that targeted various consumers of healthcare (*e.g.*, patients, hospitals, and private payers), including the following:

• the number of providers at each center
• whether there were data available for each provider
• the number of patients reported
• the number of all wounds reported
• the mean wound healing rate (%) for all wounds
• time-to-heal (weeks) for all wounds
• the number of all DFUs reported
• the mean wound healing rate (%) for DFUs
• time-to-heal (weeks) for DFUs
• the severity of DFUs
• whether or not adverse events were reported (including amputations, infections, and hospitalizations)
• whether or not data censoring rules were reported
• whether or not risk stratification was used.

We also checked the corresponding Facebook pages of each clinic for posts related to wound healing data. Because Google truncates the search items, we did not find a representative clinic from all 50 states. We next searched “wound center healing rates,” which resulted in 8,120,000 websites, but the same results were provided as with the initial search. We found wound healing rates published online from clinics in 35 states. For the remaining 15 states, we then searched wound clinic healing rates state-by-state for Alaska, Hawaii, Oregon, Arizona, New Mexico, Colorado, Utah, North Dakota, South Dakota, Oklahoma, Louisiana, Mississippi, Georgia, Virginia, and Tennessee. Data were available for 44 clinics in 44 states. There were no clinics with healing rates published online in South Dakota, North Dakota, Utah, Oregon, Hawaii, and Alaska.

Clinic and wound healing data from 44 entities are summarized in Table 1. Reported healing rates were very high for all wounds (at least 80%). Among the 40 centers that provided the percent of wounds healed, 34 (85%) reported healing rates of at least 90% (mean: 92%; standard deviation [SD]: 4.5). Time-to-heal (provided as a mean or median) was available for 30 clinics and varied from 2.7 to 16 weeks, with the majority reporting healing within 4.3 weeks (16; 53.3%).

**Table 1.** Publicly reported online healing rates and related data by wound centers in the United States (n = 44)

*Facility(-ies)*	*City(-ies)*	*State*	*Year(s) Data Reported*	*No. of Providers*	*Total No. of Patients Reported*	*Total No. of Wounds Reported*	*Mean Wound Healing Rate (%)*	*Time to Heal (Weeks)*	*References*
Fremont Health Center for Wound Healing^a^	Fremont	NE	2016 for 2015	5	N/A	N/A	97%	4 (Median)	^9^
Hoag Wound Healing and Hyperbaric Medicine Center/Hoag Health Center Irvine—Sand Canyon^a^	Newport Beach, Irvine	CA	2016	5	N/A	N/A	98%	6 Days faster than national average	^10^
The Wound Healing Center at Missouri Baptist^a^	St. Louis	MO	2016	N/A	600^b^	N/A	750 Healed wounds^b^	3.6 (Median)^b^	^11^
Center for Wound Care & Hyperbaric Medicine at Sharon Hospital^a^	Sharon	CT	2016	9	N/A	N/A	>98%	N/A	^12^
The Wound Center at Bucyrus Hospital	Bucyrus	OH	2016	5	N/A	N/A	>90%	3^b,c^	^13^
The Center for Wound Healing and Hyperbaric Medicine Doctors Community Hospital^a^	Lanham	MD	2012	3	N/A	N/A	96%	N/A	^14^
The Wound Care Center at Portsmouth Regional Hospital^a^	Portsmouth	NH	2017	N/A	>5,000	N/A	>90%	Treatment lasts 8–10 weeks	^15^
The Wound Healing Center of Amery Regional Medical Center^a^	Amery	WI	2015–2017	16	N/A	N/A	81% Within 14 weeks; 92% of all chronic wounds^b,d^	Most wounds heal within 14 weeks	^16^
The Wound Care Center at Flushing Hospital Medical Center^a^	Flushing	NY	2014	N/A	N/A	N/A	91%	N/A	^17^
The Center for Wound Healing at Bayshore Community Hospital^a^	Holmdel	NJ	2017	N/A	N/A	N/A	>95%	N/A	^18^
The Anna Jacques Hospital Wound Healing and Hyperbaric Center	Newburyport	MA	2017	7	N/A	N/A	95%	N/A	^19^
Wyoming Hyperbaric & Wound Treatment Center^a^	Casper	WY	2015	N/A	N/A	197 Healed	96%	3 (Median)	^20^
Wound Healing Center of Heart of Lancaster Regional Medical Center^a^	Lancaster/Lititz	PA	2017	4	N/A	N/A	95%	N/A	^21^
Wound Care Center at Hunt Regional Medical Center at Greenville^a^	Greenville	TX	2012^b^	N/A	N/A	N/A	94%^b^	N/A	^22^
Conway Regional Health System Wound Healing Center	Conway	AR	2017	4	N/A	N/A	89% Healed at 16 weeks	N/A	^23^
The Wound Care Center at Rush Oak Park Hospital^a^	Oak Park	IL	2015	8	N/A	N/A	>95%	N/A	^24^
Wilson Wound Healing Center^a^	Wilson	NC	2014	4	N/A	N/A	96%	4	^25^
Wound Care Center at St. Catherine Hospital^a^	Garden City	KS	2013	N/A	>7,000; >50,000 Encounters	N/A	94%	N/A	^26^
St. Luke's Wound & Hyperbaric Center^a^	Hiawatha	IA	2017	6	N/A	N/A	>90%	N/A	^27^
The Wound Care Center at Northern Nevada Medical Center^a^	Sparks	NV	2016	N/A	N/A	N/A	91%	4.3	^28^
Wound Healing Center at Allegen General Hospital^a^	Allegan	MI	2017	N/A	N/A	N/A	89% Healed at 16 weeks	N/A	^29^
South County Health Wound Care Center^a^	Wakefield	RI	2013	8	500 Patients; 3,947 encounters	N/A	At least 91%	Within 4.3 weeks	^30^
Leesburg Regional Medical Center Wound Care & Hyperbaric Center^a^	Leesburg	FL	2012^b^	N/A	1,619	N/A	>90%	4.6	^31^
Paul B. Hall Wound Healing Center^a^	Paintsville	KY	2012	N/A	580 for 2011	N/A	91%	Within 4.3 weeks	^32^
Baptist Easley Wound Care & Hyperbaric Center^a^	Easley	SC	2015	N/A	N/A	N/A	At least 91%	Within 4.3 weeks	^33^
DCH Wound Healing Center^a^	Tuscaloosa	AL	2017	N/A	N/A	N/A	80%	Within 12–16 weeks	^34^
The Wound Care Center at Providence Medical Group	Missoula	MT	2017	4	N/A	N/A	>85%	N/A	^35^
The Weirton Medical Wound Treatment Center	Weirton	WV	2017	N/A	N/A	N/A	98%	N/A	^36^
Ridgeview Medical Center Wound & Hyperbaric Healing Center^a^	Waconia	MN	2017	7	N/A	N/A	97%	Within 14 weeks	^37^
Cascade Valley Hospital Wound Care & Hyperbaric Medicine Center^a^	Arlington	WA	2013	N/A	380	N/A	At least 91%	Within 4.3 weeks	^38^
Brattleboro Memorial Hospital Center for Wound Healing^a^	Brattleboro	VT	2013	4	>200	N/A	96%^b^	Within 4.3 weeks	^39^
Southern Maine Health Care Wound & Ostomy Care Center	Biddeford	ME	N/A	N/A	N/A	N/A	N/A	Mean 7.8 weeks	^40^
Portneuf Wound Care and Hyperbaric Center^a^	Pocatello	ID	2015	9	N/A	N/A	93%	Within 4.3 weeks	^41^
Fauquier Health Wound Healing Center^a^	Warrenton	VA	N/A	5	N/A	N/A	93%	Within 4.3 weeks	^42^
Johnson Memorial Hospital Wound Healing Center^a^	Franklin	IN	2012^b^	N/A	N/A	N/A	>91%	Within 4.3 weeks	^43^
Bayhealth Wound Care Center^a^	Dover	DE	2014	6	N/A	N/A	At least 91%	Within 4.3 weeks	^44^
Yavapai Regional Medical Center's Advanced Wound Care Center	Prescott Valley	AZ	2016	N/A	N/A	N/A	80%	Within 4 weeks	^45^
Memorial Medical Center Wound Care Center^a^	Las Cruces	NM	2015^b^	N/A	400	N/A	At least 91%	Within 4.3 weeks	^46^
Penrose-St. Francis Wound Care Clinic	Colorado Springs	CO	2017	3	N/A	N/A	N/A	Mean 6.4 weeks	^47^
Center for Wound Care & Hyperbaric Medicine at Comanche County Memorial Hospital^a^	Lawton	OK	2014^b^	N/A	3,500 from 2004–2014	7,000 from 2004–2014	At least 91%	Within 4.3 weeks	^48^
St. Tammany Parish Hospital Clinic for Wound Care and Hyperbaric Medicine	Covington	LA	2015	N/A	N/A	N/A	N/A	Mean 4.7 weeks	^49^
King's Daughters Wound Healing Center^a^	Brookhaven	MS	2017	N/A	N/A	N/A	>81%	83% Within 16 weeks	^50^
Mary Washington Healthcare Wound Healing Center^a^	Fredericksburg	VA	2015^b^	N/A	8,400 Specialized treatments	N/A	At least 91%	Within 4.3 weeks	^51^
Hardin HMC Wound Care Services^a^	Savannah	TN	2012	N/A	N/A	N/A	98%	2.7 Weeks	^52^

^a^ Healogics facility; ^b^data obtained from posts on Facebook; ^c^time-to-heal data reported for 2012 only; ^d^healing rates reported for 2015 only.

N/A, not available.

Although 20 clinics (45.4%) listed the number of providers, there were no data provided per individual provider. Only 10 clinics (22.7%) reported the total number of patients, and only 2 clinics (4.5%) reported the total number of wounds. Penrose-St. Francis Wound Care Clinic in Colorado Springs, CO was the only clinic to provide a mean time-to-heal for DFUs (11.1 weeks), in addition to the mean time-to-heal for all wounds (6.4 weeks).^47^ No other clinic provided DFU-related data. No clinics reported adverse events, data censoring rules, or the use of risk stratification, although The Center for Wound Healing and Hyperbaric Medicine Doctors Community Hospital in Lanham, MD, clarified that their data were based solely on patient adherence.^14^

### A “different reality” of wound healing rates reported by RCTs

We next utilized RCT data to establish the most optimistic wound healing rate possible.

We previously established that RCTs in wound care, almost without exception, exclude patients with significant comorbid diseases to evaluate the efficacy of the study agent, resulting in the ineligibility of more than half the wound care patient population.^53^ Virtually all prospective trials in wound healing are designed to allow wound epithelialization within 12–16 weeks and thus select a less sick patient population with relatively small, superficial ulcers. We recently confirmed this finding when comparing recent RCT data with the real-world patient data of a wound care research consortium.^54^ We found that the mean size of VLUs and DFUs of the consortium patients were, respectively, five and three times the size of the ulcers enrolled in the consortium RCTs. Furthermore, 43.6% of the consortium patients with DFUs had more severe ulcers graded at Wagner 3 or higher, when only Wagner 1 and 2 ulcers were eligible for the RCT.

We performed a search of 50 RCTs on PubMed using the following search terms: “diabetic foot ulcers,” “venous leg ulcers,” and “pressure ulcers” through February 1, 2017. We reviewed abstracts and selected articles that analyzed healing rates at 12 weeks for control/placebo groups. We collected data on the number of patients, mean age (years), the number of wounds, wound severity, mean initial wound area, whether or not ischemia was allowed, which comorbidities were allowed, the percent of wound healed at 12 weeks, and the time to heal.

Our literature search returned 48 RCTs meeting our search criteria, including 20 VLU trials, 26 DFU trials, and 2 pressure ulcer (PU) trials. There were 2,620 control subjects with 2,624 study wounds enrolled in all 48 RCTs. The data from these trials' control groups are summarized in Table 2. The wound healing data contrasted strikingly with the data reported online by wound centers. The mean wound healing rate at 12 weeks among control subjects in the ideal environment of these trials was 40.0% (SD: 20.2%; range: 7.7–90.6%), at least half of the rates reported by the wound centers. The mean VLU healing rate was 42.7% (SD: 20.1%; range: 12.5–88.3%). The mean healing rates for DFU and PU trials were 37.9% (SD: 21.2%; range: 4.0–90.6%) and 40.0% (SD: 5.7%; range: 36–44%), respectively. The times to heal varied extensively for all wound trials and were provided as means or medians. The rate was as low as a mean of 5.1 weeks for a DFU trial^95^ to as high as a median of 36 weeks for a VLU trial.^55^ None of the trials had a time to heal within the 4.3 weeks reported by majority of the wound centers.

**Table 2.** Healing rates of chronic wounds reported for control groups in randomized controlled trials at 12 weeks

*Wound Type*	*No. of Patients*	*Mean Age (Years)*	*No. of Wounds*	*Wound Severity*	*Mean Wound Area (cm^2^)*	*Ischemia*	*Comorbidities Included*	*Percentage of Wounds Healed at 12 Weeks*	*Time to Heal (Weeks)*	*References*
VLU	169	70.0	169	• Mean: 1.8 VLUs on leg • Mean: 3.7 prior VLU episodes	27.2	ABI >0.8	• Mean BMI: 30.7 • Immobile: 28.6% • Mean HbA1c: <10%	20.0%	36 (Median)	^55^
VLU	46	51.5	46	• Depth: full thickness down to fascia	3.6	ABI >0.7	• Mean BMI: 24.5	28.0%	12.1 (Median)	^56^
VLU	43	57.0	43	• Median duration: 5 months	11	ABI >0.9	• Hypertension: 42% • Obese: 33%	50.0%	15.4^a^	^57^
VLU	106	72.4	106	• Bilateral ulceration included • Diameter: >1 cm	N/A	ABI >0.8	• Hypertension: 36% • Osteoarthritis: 29% • Rheumatoid arthritis: 20% • Heart disease: 14% • History of stroke: 7%	56.7%	8.3 (Median)	^58^
VLU	195	71.9	195	• Multiple ulceration included • Length/width: ≥1 cm • 59% Had prior VLU episodes • Mean duration: 3 months	3.8	ABI ≥0.8	N/A	48.7%	13.1 (Median)	^59^
VLU	58	65.0	58	• Depth: extended through the epidermis and dermis with no exposed tendon or bone • 34% Had duration >12 months	12.1	ABI ≥0.8	• Mean BMI: 30.9	34.0%	N/A	^60^
VLU	8	62.0	8	• Mean duration: 30 months	12.3	ABI >0.9	N/A	12.5%	N/A	^61^
VLU	27	61.4	27	• Multiple ulceration included • 56% Had recurrent ulcer • Mean duration: 26.7 months	12.2	ABI ≥0.8	• Mean BMI: 32.2	48.0%	6.9 (Mean)	^62^
VLU	28	67.5	28	• Area: <10 cm^2^ • Mean duration: 21 months	3.1	N/A	N/A	78.0%	N/A	^63^
VLU	20	75.6	20	• Area: ≤100 cm^2^ • ≥20% covered in slough • No exposed vessels, tendon, muscle, or bone • Mean duration: 32 months	10.8 (Median)	ABI: 0.9–1.3	• Not stated, but history of bleeding disorders excluded	73.0%	N/A	^64^
VLU	180	69.1	180	• Area: <25 cm^2^ • No exposed muscle, tendon, or bone • Clean, granulating base • Minimal adherent slough • Median duration: 10.4 months • 48% Had recurring ulcer	7.2 (Median)	ABI: 0.8–1.2	• Excluded • Mean BMI: 30.1	31.0%	N/A	^65^
VLU	181	68.3	181	• Mean Venous Clinical Severity Score: 15.0% • 2.8% Had mixed venous/arterial ulcer • Median duration: 3.7 months	2.6	ABI >0.7	• Excluded diabetes, rheumatoid arthritis, and peripheral arterial disease	49.7%	9.3 (Mean)	^66^
VLU	60	63.0	60	• Widmer stage III • CEAP 6 • 2% Had multiple VLUs	55.9	ABI >0.9	• Obesity: 32% • Diabetes: 12%	31.7%	6.6 (Mean)	^67^
VLU	33	72.9	33	• Mixed etiology permitted without maceration • Mean duration: 8.3 months	≥1 cm^2^; ≤50 cm^2^	ABI >0.8	• Diabetes: 3.6%	47.1%	N/A	^68^
VLU	29	70.8	29	• Noninfected ulcer >2 cm^2^, but <10 cm in any dimension	9.7	ABI >0.8	• 55% Had major clinical conditions present	17.0%	14.4 (Mean)	^69^
VLU	40	68.7	40	• Infected ulcer of CEAP CVI grade C6	≥2 cm^2^; ≤20 cm^2^	N/A	N/A	32.0%	12.4 (Median)	^70^
VLU	36	71.7	36	• Mean duration: 9.9 months • 38.8% Had multiple ulceration • 69.4% Had previous ulceration	9.5	ABI ≥0.8	• Diabetes: 11.1% • Obesity: 19.4% • Hypertension: 58.3% • Coronary disease: 5.6%	33.0%	N/A	^71^
VLU	22	79.3	22	• Area: ≤20 cm^2^ • Mean duration: 9.5 months	7.4	ABI ≥0.8	• Excluded • 45% Had BMI >30	46.0%	N/A	^72^
VLU	60	70.1	60	• Noninfected ulcer with viable wound bed with granulation tissue • No exposed tendon or bone • Mean duration: 18.1 months	13.4	ABI: 0.8–1.3	• Excluded • Mean BMI: 30.9	88.3%	11.4 (Mean)	^73^
VLU	31	59.0	31	• Area: ≤35 cm^2^ • Mean duration: 11 months	8.1	Significant arterial insufficiency excluded	• Excluded	29.0%	N/A	^74^
DFU	45	68.0	45	• Wagner 2: 22% • Wagner 3: 62% • Wagner 4: 11% • Previous vascular surgery: 49%	2.8	No restriction	• Mean duration of diabetes: 23 years • Hypertension: 73% • Renal impairment: 80%; prior minor amputation: 47%	4.0%	N/A	^75^
DFU	27	60.8	27	• Wagner 1 and 2	2.7	ABI >0.7	• Various comorbidities excluded (*e.g.*, renal dysfunction)	37.0%	N/A	^76^
DFU	ITT: 31; PP: 27	59.0	31 (ITT); 27 (PP)	• Wagner 1 and 2	1.8	ABI >0.7	• Excluded	47.0%	8 (Mean); 9 (median) for PP only	^77^
DFU	21	55.9	21	• UT Grade 1 • Mean volume: 1.0 cm^3^	3.6	Excluded	• Excluded	42.9%	12.1	^78^
DFU	39	60.6	39	• Full-thickness neuropathic ulcer • Mean duration: 20.4 months	3	Excluded	• Excluded	26.3%	Unable to determine, because <50% of ulcers healed	^79^
DFU	13	53.8	13	• Full-thickness ulcer of the plantar surface or heel free of infection • No exposed tendon, bone, or joint • Mean duration: 4.6 months	1.9	Doppler AAI >0.7	N/A	7.7%	>12 Weeks (median)	^80^
DFU	22	58.2	22	• Noninfected ulcer extending through the dermis and into subcutaneous tissue • No exposed muscle, tendon, bone or joint • Area: <20 cm^2^ • Mean duration: 18.6 months	1.5	Palpable pulse present; AAI >0.7	• Excluded • Mean BMI: 32.6	14.3%	>12 Weeks (median)	^81^
DFU	47	≥65 Years: 27.7%	47	• Noninfected ulcer • Area: 1–15 cm^2^ • No exposed muscle, tendon, bone, or joint • Mean duration: 4 months	3.9	ABI: ≥0.7 or <1.3	• Mean HbA1c: 7.8 • Mean BMI: 25 • Mean albumin: 4.0	21.0%	9.9	^82^
DFU	115	55.5	115	• Noninfected ulcer extending through the dermis and into subcutaneous tissue • No exposed muscle, tendon, bone or joint • Area <20 cm^2^ • Mean duration: 15.5 months	2.5	Doppler AAI ≥0.7	N/A	18.0%	Median percent wound closure was 78% by week 12	^83^
DFU	20	61.1	20	• UT Grade 1A or 2A	3.9	ABI ≥0.9	• Excluded	85.0%	6.7	^84^
DFU	35	60.6	35	• No exposed tendon, muscle, capsule, or bone • Area: <25 cm^2^ • Mean duration: 3.2 months	3.1	TcPO_2_ > 30 mmHg; ABI: 0.7–12.1	• Hypertension: 74.3% • Coronary artery disease: 28.6% • Congestive heart failure: 8.6% • Mean BMI: 34.7	51.0%	4.8	^85^
DFU	19	64.4	19	• Wagner 1 and 2 • Mean duration: 2.8 months	3.5	ABI ≥0.7	• Mean BMI: 25.7 • Serum creatinine >2 mg: 15.8% • 89.6% Had hypertension, coronary heart disease, and/or hyperlipidemia	42.0%	N/A	^86^
DFU	96	56.0	96	• Full-thickness neuropathic ulcer • Area: <17 cm^2^ • Mean duration: 11.1 months	2.8	ABI ≥0.7	• Excluded • Mean BMI: 33.1	38.0%	12.8 (Median)	^87^
DFU	138	59.0	138	• Wagner 1 and 2 • Mean duration: 3 months	3.1	Excluded	• Excluded	28.3%	5.8 (Mean)	^88^
DFU	153	57.3	153	• Noninfected full-thickness neuropathic ulcer • No exposed capsule, tendon, or bone, • Area: <12 cm^2^ • Mean duration: 10 months	3.7	ABI: 0.7–12.1; TcPO_2_ > 40 mmHg	• Excluded • Mean BMI: 34.1	32.0%	6.5 (Median)	^89^
DFU	39	58.9	39	• UT Grade 1 or 2 • <25 cm^2^ • Mean duration: 5.3 months	5.1	TcPO_2_ ≥ 30 mmHg; ABI: 0.7–12.1	• Excluded • Mean BMI: 34.6	46.2%	6.8 (Mean); 7.0 (median)	^90^
DFU	24	56.8	24	• Wagner 1 and 2	1.9	ABI ≥0.8	• N/A • Mean BMI: 35.5	33.0%	23 (Median)	^91^
DFU	28	60.0	28	• Wagner 1–3 • Superficial infection: 66.7% • Deep infection: 16.7% • Mean duration: 7.5 months	5.4	ABI: 0.7–12.1	• Hypertension: 12.5% • Dyslipidemia: 33.3% • Ischemic heart disease: 45.8% • Cerebrovascular disease: 45.8% • Severe renal/heart disease and life-threatening primary diseases excluded • Mean BMI: 24.4	37.5%	10.7	^92^
DFU	58	64.1	58 Diabetic ulcers; 55 DFUs	• Wagner 2–3 • Median duration: 0.9 months	2.9	ABI ≥0.6	• Excluded • Albumin/globulin: 1.1 • Creatinine: 91.1 • Mean HbA1c: 9.8	67.3% (for DFUs)	6.4 (Median; for all diabetic ulcers)	^93^
DFU	33	67.8	33	• Noninfected DFU • Duration: >3 months	15.7	Mean ABI: 0.9	• Mean BMI: 31.7 • Hypertension: 69.7% • Chronic venous insufficiency: 42.4% • Dyslipidemia: 33.3% • Ischemic heart disease: 12.1% • Cerebrovascular disease: 9.1% • Heart failure: 9.1% • Previous peripheral revascularization: 21.2%; • Intermittent claudication: 36.4% • Previous amputation: 36.4%	33.3%	N/A	^94^
DFU	32^b^	60.6	32	• Multiple ulceration included • UT IC: 65.6% • 1A: 28.1% • 1D: 6.3%	N/A	Ischemic: 68.8%; mean ABI: 0.9	• Mean BMI: 27.4 • Previous ulceration: 65.6% • Previous amputation: 9.4% • Other clinical conditions excluded	90.6%	5.1 (Mean)	^95^
DFU	80	62.0	80	• Wagner 1 and 2 • Mean duration: 7.3 months	6.7	ABI ≥0.5; TcPO_2_ ≥ 20 mmHg	N/A	21.0%	8.3 (Mean)	^96^
DFU	26	62.4	26	• Wagner 1: 35% • Wagner 2: 65% • Mean duration: 4.8 months	5.2	TcPO_2_ ≥ 40 mmHg	• Excluded • Mean BMI: 22.8 • Mean HbA1c: 7.5 • Albumin: 3.9	69.0%	8.1 (Median)	^97^
DFU	32	63.8	32	• Wagner 1: 26% • Wagner 2: 74% • UT 1A: 25% • 2A: 34% • 3A: 41% • Mean duration: 6.2 months	2.9	TcPO_2_ ≥ 30 mmHg	• Excluded • Mean BMI: 23.2 • Mean HbA1c: 7.4 • Mean albumin: 3.9	34.0%	6.9	^98^
DFU	20	67.0	20	• Deep ulcer extending the muscle, tendon, or bone: 65% • Superficial ulcer extending through the full thickness of dermis: 35%	1.5	Systolic toe press <45 mmHg	• Excluded • Mean HbA1c: 7.7 • Mean diabetes duration: 18 years • Retinopathy: 30%	25.0%	N/A	^99^
DFU	20	59.9	20	• UT Grade 1A and 2A • Mean duration: 5.5 months	N/A	TcPO_2_ > 30 mmHg	• Mean diabetes duration: 17.0 years • Mean HbA1c: 9.5 • Neuropathy: 100%	35.0%	6.9	^100^
PU	22	77.9	22	• Stage III and IV	4.1	Vascular conditions included	• Controlled diabetes included • Infection excluded	36.0%	25.7^c^	^101^
PU	20	77	20	• Stage III and IV truncal PUs	12.1	N/A	• Excluded infection and patients with venous, arterial, and/or diabetic ulcers	44.0%	N/A	^102^

^a^ Estimated for 75% of patients; ^b^25 patients enrolled in both study groups with 64 ulcers; 32 ulcers were allocated to the control group, with each ulcer counting as 1 participant; ^c^extrapolated mean closure time.

AAI, ankle-arm index; ABI, ankle brachial index; BMI, Body Mass Index; CEAP, Clinical severity/Etiology or cause/Anatomy/Pathophysiology; CVI, chronic venous insufficiency; DFU, diabetic foot ulcer; HbA1c, glycated hemoglobin; ITT, intention-to-treat population; PP, per-protocol population; PU, pressure ulcer; TcPO_2_, transcutaneous partial pressure of oxygen; UT, University of Texas; VLU, venous leg ulcer.

What is even more alarming about the difference in healing rates between the RCTs and the wound centers is that we know many of the RCTs excluded patients with significant clinical comorbidities and ischemia, and the more severe wounds. It is reasonable to assume that the subjects enrolled in these RCTs were less sick than real-world patients and still, their healing rates were drastically lower that those reported online. It is likely that in the real world, among complicated patients (many of whom suffer from serious wounds and comorbidities), healing rates better than 40.0% are not, in fact, actually achievable.

### Evaluation of real-world quality data: saying what we mean

To determine true wound healing rates, we cannot apply RCT exclusion criteria, because we need to understand and include all real-world patients. Patients with chronic wounds are older and very sick.^54,103^ Our previous research of USWR real-world data demonstrated that, if all patients are reported, the national wound healing rate is ∼66%.^103,104^ We next evaluated uncensored real-world data from the USWR.

The general methodology for obtaining datasets suitable for wound care analysis has been previously described.^103,105^ The dataset analyzed included all DFUs, VLUs, and PUs with in-service visit dates from September 28, 2001, through December 1, 2016, comprising 71,957 DFUs, 77,891 PUs, and 99,588 VLUs. To account for one-time (consultation) visits and patients whose wounds were still in service (and thus without outcomes), 17,662 wounds that only had 1 visit and 11,447 wounds that were still in service were deleted from the dataset leaving 62,964 DFUs, 66,577 PUs, and 97,420 VLUs for analysis. Outcomes were determined for percentage of wounds healed at 12 weeks, using a window of ±3 days around 84 days after the first clinic visit in which the wound was examined, and percentage of wounds that ever healed without time limit. The algorithm employed to determine whether the wound was healed has been previously reported.^105^ The results show that at 12 weeks, about 30% of DFUs and PUs were healed, whereas nearly 45% of VLUs were healed (Table 3). Without time constraints, substantially more wounds were healed, but VLUs have the highest percentage of wound closure by wound type at 56.9% healed.

**Table 3.** Percentage healing rates for the three most common types of chronic wounds at 12 weeks and regardless of time with mean follow-up times, based on data from the U.S. Wound Registry

*Time Period*	*DFUs*	*PUs*	*VLUs*
12 Weeks	30.5	29.6	44.1
No period of time specified	45.1	43	56.9
Mean follow-up time in weeks (SD)	19.7 (36.17)	24.5 (48.97)	16.1 (33.56)

SD, standard deviation.

## Discussion

### The “war against reality” and the casualty of the honest wound healing rate

When it comes to outcome data, both providers and consumers should be cautioned that the internet is currently a maddening Wonderland. Without a standardized risk stratification method, there is considerable pressure to inflate healing rates on websites and social media accessed by consumers, because not to do so will make the practitioners attempting accurate reporting appear less clinically capable.^103^ Consequently, data are vetted by reclassifying patients with wounds that do not heal under palliative or complex care, thereby removing these patients from the denominator for public reporting. Similarly, patients who do not return to the clinic after 30 consecutive days are reported as lost to follow-up and removed from the dataset.^106^

Wound healing rates publicly reported online and directed at healthcare consumers consistently listed rates of 80% or better (mean: 92%), with the majority reporting. No reporters provided transparency of data censoring practices or included a discussion of adverse event rates. Indeed, none of these entities acknowledged that poor outcomes ever occurred. Also of importance is that not a single center explained how it defined a healed wound. The lack of clearly defined wound outcomes is a challenge to data reporting.^105,107^ The U.S. Food and Drug Administration (FDA) defines a healed wound as reepithelialized skin without drainage or dressing requirements confirmed at 2 consecutive visits 2 weeks apart,^108^ yet in a review of 176 articles reporting wound outcomes, 19% did not provide a clear definition of a healed wound.^107^ Ultimately, “healing” may not be the ideal measure for quality reporting given the fact that real-world wounds may require many months to accomplish complete closure. It may be that intermediate outcomes can be identified which are better. It is important to reach a consensus regarding patient exclusions, as further explained at the end of this section. For example, the Wound Healing Index (WHI) is currently available for seven major wound types. Wounds that do not fit one of these categories will have to be excluded from reporting since it will not be possible to stratify them across different sites. A more general risk stratification system could likely be developed for the less common ulcer types (*e.g.*, sickle cell ulcers), if funding were available, but given the general lack of investment in wound healing research, additional risk models are not on the horizon. It should be noted that an online Google search for wound healing rates does not produce random results, but rather uses industry ranking and device-specific algorithms that are tailored to the consumer preferences of each device using Google. Therefore, the selected 490 search results provided involve a degree of selection bias. The consistently high healing rates reported online can be attributed to the fact that most of the centers (35/44; 79.5%) identified by our online search are Healogics facilities, which target a national healing rate of 92%.^21^ Based in Jacksonville, FL, Healogics, Inc. is the largest for-profit wound care operator in the United States, with nearly 800 affiliated facilities and more than 3 million wounds treated.^109^ Healogics centers strive for a patient satisfaction rate higher than 92% and a healing rate of at least 91% in under 30 median days.^110^ These healing rates are unachievable in the absence of data censoring rules designed to exclude patients solely because they did not heal, a statistical method truly worthy of Lewis Carroll.

All publicly reported information also indicated that healing would be achieved within a period of time similar to those seen in clinical trials (*e.g.*, 12 weeks), with the majority of entities citing a time to heal of within 4.3 weeks. We do not, however, know what reporting timeframe was used. While 12 weeks is the recommended follow-up period for a clinical study, this timeframe should vary by wound type and other patient and wound characteristics.^111^ It is important to note that some facilities reported mean times to heal, others reported median times to heal, and some did not specify. This heterogeneity in reporting time to heal was also demonstrated by the RCTs analyzed and is a common issue that renders clinically relevant comparisons impossible.^112^ Another issue that complicates the quality and integrity of time-to-heal data is that they are usually reported from outpatient wound clinics, with few patients requiring hospitalization and even fewer requiring subacute care, and do not necessarily encompass the entire episode of care.^106^ For example, Ennis *et al.* reported a 5-week time to heal of a VLU treated at an outpatient clinic.^106^ However, the entire continuum of care lasted 69 weeks, when they counted prior care in the primary care setting, a wound clinic, home healthcare, subacute care, and hospitalization. The authors further pointed out that when the same clinical team followed and provided the same wound management program in both the outpatient and subacute settings, the outpatient healing rates were 72–74%, whereas only 41.6–45.9% of patients healed in the subacute programs.^113^ Importantly, only 10% of their patients were admitted for subacute care; these were sicker and more complex patients who could not be expected to have healing rates similar to those treated at an outpatient wound clinic.

To improve the reporting of outcomes that better reflect the entire episode of care and reduce the effect of the clustering of observations (when patients are treated at multiple sites of care and/or by multiple providers),^105^ providers need to improve the reporting of what happens to patients if they stop visiting their particular clinic. There are a variety of reasons that patients are not counted in wound healing denominator calculations, with patients

• Only visiting the clinic for consultations, not treatment
• Lost to follow-up
• No longer visiting the clinic after 1 or 2 months
• Moving outside the clinic's geographical coverage area
• Transferred to another facility or care setting (*i.e.*, hospital, acute care, long-term care, nursing home, and home healthcare).
• Dying, which can occur especially among patients with more severe DFUs and comorbidities
• Simply deciding to no longer return to the clinic and/or continue care.

Wound outcome data absolutely must consider the site and setting of care, the wound management and standard of care undertaken, the point at which providers are involved in the continuum of care, and risk stratification of patients and wound complexity and severity to report honest rates.^106^

RCTs of uncomplicated, small ulcers of different wound types among relatively healthy subjects consistently reported mean healing rates around 40% (Table 2), although these rates varied widely across trials (range: 7.7–90.6%). Based on uncensored USWR data (Table 3), the healing rate of DFUs among typical patients at hospital-based outpatient wound centers may be as low as 30.5%. Based on real-world data and RCTs, healing rates over 90% as publicly reported can be achieved only by creating extreme censoring rules, which are not likely to fall within acceptable standards of data management, but can be summarized as, “We are all mad here.” Truth is not the only casualty of this system. By reporting that nearly all wounds heal, we are unable to elucidate the relative contribution of specific interventions, many of which are being called into question on the eve of capitated or episode-based payments. Indeed, inflating healing rates makes it is nearly impossible to develop episode-based payment models, a task upon which the future survival of the field of wound care may depend.

How shall we reconcile these diverse observations to allow for useful reporting of wound healing rates and other quality measures? Inflated healing rates accessed by consumers online are for marketing purposes only and are not reported to CMS or any other quality organization. Both CMS and the National Quality Forum (NQF) work diligently to standardize quality measures. CMS requires that duplicative or overlapping measures undergo “measure harmonization,” which requires the measure sponsors to work collaboratively to resolve differences and develop a single measure to be implemented.^114^ However, CMS has not developed any quality measures focused on wound outcomes at the national level. QCDRs were intended to allow specialty societies to fill measure gaps. Since wound care is not a recognized medical specialty, it lacks a consensus-forming body. The USWR has tried to fill the measure gap in wound care by creating the WHI and developing outcome measures. Success has been limited, because, under MIPS, practitioners may choose which quality measures they wish to report and have no incentive to tackle the challenge of risk-stratified outcome reporting when easier (although less relevant) measures suffice.

Therefore, to improve the transparency of the public reporting of data and transform healthcare culture, the questions are as follows: who should publicly disclose quality data and patient information and how should it be done?^8^ USWR data suggest that rather than reporting unbelievably inflated healing rates, or reporting uncensored data with so many confounding factors as to be uninterpretable, some middle ground is possible to achieve quality measure reporting. It is possible to use survival approaches to wound healing outcomes that include right censoring of patient wound data. Based on the USWR data reported herein, it seems reasonable to exclude from outcome reporting the following wounds:

• Wounds in patients transferred to another clinic or setting for treatment so that their outcome is not known; however, the challenge here is that sicker patients with more complex wounds are more likely to be transferred to an acute care facility because of complications
• Wounds in patients who are lost to follow-up, if their final outcome is not known
• Wounds in patients who make fewer than three visits within some clinically relevant timeframe (*e.g.*, 4 weeks).

Wounds in patients who die in service may need to be reported as having the adverse event of death, although further discussion on this point is warranted. Although provider performance should not be judged by the outcome of patients they did not finish treating, nearly complete wound healing is commonly observed for many patients before death. Public reporting should also evaluate provider performance based on their caring of the sickest patients. More transparent and honest wound healing outcome data will follow after the quality of care is measured based on the percentage of healed patients when risk stratification is used to determine their healing likelihood.^115^ By using risk stratification, we could identify which practitioners or institutions may be providing exemplary care, not by reporting healing rates over 90%, but by a healing rate of, for example, 50% among wounds with only a 30% predicted likelihood of healing. A study is underway that will use risk stratification to assess provider performance.

### Criteria to reporting real-world quality data

We recently published the “ABCs of Registries,” a list of reporting standards for publications of real-world wound registry data to minimize the sources of bias.^103^ These criteria are based on the collection of all patient and wound data at the point of care to be transmitted directly to an electronic health record, which implements a risk stratification model that creates matched cohorts for different wound types. We also published 13 guidelines based on the RECORD statement to improve the reporting of wound care analyses derived from electronic health records and registries to minimize biases and make more realistic comparisons of the results and outcomes.^105^ Similar criteria are also needed for the public reporting of real-world data. The majority of our guidelines for reporting wound care analyses are also applicable to public reporting of outcome data. In Table 4, we have summarized the criteria needed to publicly report wound healing rate data based on the current limitations demonstrated by this study.

**Table 4.** The current limitations to publicly reporting wound healing rates and the criteria needed to report honest healing rates

*Limitation*	*Criteria to Report Honest Wound Healing Rates*	*Comments*
(1) Lack of standardized definitions for wound outcomes^105,106,113^	Healed wound = completely closed wound confirmed by two visits 2 weeks aparts.^105^	Amputations are considered nonhealed wounds.^105^
(2) Lack of timeframe (by wound type)^103,105,106^	Healing rates to be reported based on percent healed at 1 year; time to heal to vary by wound type.^103,105^	The time to heal of a DFU may be based on 3–6 months, whereas a VLU may be based on 1–2 years.
(3) Variation in diagnostic codes across wound types^103,105^	Define wound types.	Because wounds are symptoms of an underlying disease, they often refuse neat categories. Many patients are on immunosuppressives, many patients with leg ulcers have both venous and arterial disease; 33.1% of patients with chronic wounds that are not DFUs have diabetes.^104^ Diagnosis is difficult.^105^
(4) Standard of care and advanced therapy (as applicable) are not defined	Define wound care protocols.^105,106^	Healing rates at wound centers can be delayed when proper standard of care is not utilized, which reflects poorly on provider performance. In 2009, USWR data demonstrated that only 6% of patients with DFUs and 17% of patients with VLUs receive adequate, respective standard of care of offloading and compression bandaging.^116^ These rates improved by 2015, with 56% of DFUs adequately offloaded and 88.7% of VLU adequately compressed at each visit.^117^
(5) Lack of key wound, patient, and healing factors	Include wound area, wound severity wound duration, patient age, presence of ischemia, comorbidities, and adverse events.^103,105^	These variables are used in risk stratification.
(6) Lack of risk stratification for patients and wounds	Need to report whether any risk stratification and/or severity indices was used for patients and wounds and identify model used.^103,105,106^	Providers will be more motivated to report honest healing rates when they are based on the patient's likelihood of healing and not just on the proportion of wound healed.
(7) Lack of data censoring rules	Need to report patients/wounds not included in the wound healing rate denominator.^103,105^	Providers must do a better job of tracking patients who no longer return to clinic. In the future, wound registries that could be integrated into the Medicare dataset would have mechanisms in place to track patients across sites of care.
		Patients may also have multiple wounds that are not all counted in the healing rates.
(8) Clustering of observations are pervasive with healing rates only reported by 1 site of care and may not reflect the entire continuum of care (care at multiple sites, by multiple providers, etc.).^105,106^	Need to report the healing rate based on the point along the patient's entire episode of care.^106^	Same comment as in No. 7.
(9) Lack of stratification by productivity and experience of wound care center.^106^	Need to report the annual number of patients and wounds treated, the number of providers/facility, and data by provider.	High volume and specialized centers will have weighted healing rates compared to low volume, less experienced centers.^106^

USWR, U.S. Wound Registry.

### Conclusions

The transition of Medicare physician payment to an entirely new structure in 2017 went almost unnoticed by healthcare professionals, perhaps, in part, due to all the other simultaneous cultural and political upheavals. In addition, CMS eased the transition with a “Pick Your Pace” program that allows practitioners to protect their 2017 Medicare Part B payments by reporting (*e.g.*) only one quality measure, postponing the reality of this new program until 2018.^118^ Although the focus of this article is not the arcane details of MIPS, fully implemented, wound care practitioners are not likely to be successful without utilizing measures that are relevant to their practice. In 2016, the USWR set national benchmark rates for offloading of DFUs, compression of VLUs, and arterial screening of leg ulcer patients, the components of its “Do the Right Thing^™^” initiative.^117^ CMS has given the USWR permission to publicly report physician performance data for these three quality measures on the USWR website. Physicians have begun to report to CMS the data on risk-stratified DFU and VLU outcome measures, and the USWR will begin public posting of national wound healing rates once outcome measure data have met CMS reporting standards. On the eve of public reporting of patient outcomes like wound healing rates, we must abandon the uncommon nonsense of a national healing rate of 92%, which cannot be reasonably believed, based on the healing rates documented in prospective trials (Table 2). Some data censoring is necessary to account for numerous factors, including patients who are lost to follow-up. After censoring, wounds must (and will) be risk adjusted as a matter of federal law, if clinicians wish to use relevant QCDR measures to satisfy MIPS/PQRS reporting requirements and thus avoid cuts in Medicare Part B payments. This is the only approach that can identify outliers among providers in either direction (superior or inferior), establish the need for certain advanced therapeutics, or provide justification for high resource use patients.

While wound risk adjustment continues to require more research, provider data must now be reported on Physician Compare. Many other reporting parameters should be decided, preferably by consensus, and most logically facilitated through the Alliance of Wound Care Stakeholders in similar process as were the USWR quality measures. Some basic rules for publicly reporting data have been proposed in this article, but others are needed, including the minimum number of wounds in each category to allow reporting. It is also hoped that the Wound-Care Experts/U.S, FDA-Clinical Endpoints Project currently underway will identify metrics other than wound healing or closure, which can be formulated into valuable wound outcome quality measures.^119^ During the first phase of this ongoing study, 628 wound experts and researchers identified 15 potential endpoints that will be studied further in the research phase to provide evidence supporting their use in regulatory decision-making. From these endpoints, there are four potential primary outcomes that have been content validated as both clinically relevant and patient centered: reduced amputation, reduced economic burden, improved function and ambulation, and improved quality of life.^119^ Ironically, in 2017, CMS rejected the USWR “Patient Reported Wound related Quality of Life” QCDR measure, which had been reported by only one physician in the United States in 2016, in part due to the uncompensated cost of implementing a patient-reported measure, revealing the wide gulf between those outcomes we say we value and those we are actually willing to support. Which way we go from here does depend on where we want to go. If the wound care community wants to survive healthcare reform, then it will be through risk adjustment and a transparent way of reporting wound healing rates, and other meaningful wound outcomes, including patient-reported outcomes. In the absence of federal investment in this vexing problem, it is in the best interest of manufacturers to fund improvements in the public reporting of outcomes through quality measures, since this is the way by which the value of therapeutic interventions can best be understood in the real world. When it comes to the reporting of healing rates, we agree with *Alice*, “It would be so nice if something made sense for a change.”

## Summary

In this review, we compare real-world healing rates from the USWR, a QCDR, with RCTs and publicly reported data and develop criteria for honest reporting of wound outcomes, a requirement of CMS under the new Quality Payment Program. We demonstrate how real-world and RCT data provide convincing evidence that most patients (55–70%) do not heal their wounds, in contrast with a mean publicly reported healing rate of 92%. It is likely that in the real world, among complicated patients, healing rates better than 40.0% are not achievable.

By federal law, if quality data are reported by any QCDR, at least one outcome measure must be reported and must be risk adjusted. Without a standardized risk stratification method, there is considerable pressure to inflate healing rates on media accessed by consumers, because *not* to do so will make the practitioners attempting accurate reporting appear less clinically capable. Some data censoring is necessary to account for numerous factors, including patients who are lost to follow-up. However, wounds must (and will) be risk adjusted, if clinicians wish to use relevant QCDR measures to satisfy reporting requirements and thus avoid cuts in Medicare Part B payments. By reporting that nearly all wounds heal, we are unable to elucidate the relative contribution of specific interventions, many of which are being called into question on the eve of capitated or episode-based payments. If the wound care community wants to survive healthcare reform, then it will be through risk adjustment and a transparent way of reporting wound healing rates and other meaningful wound outcomes, including patient-reported outcomes.

Take-Home Messages

• Wound outcomes must be reported under the new Quality Payment Program, and when quality data reported are by a QCDR, at least one outcome measure must be reported and risk adjusted.
• A risk-stratification tool alleviates the pressure practitioners face inflation in their healing rates, by preventing those who care for the sickest patients from being penalized by appearing to have poorer outcomes than their peers.
• While wound care provider entities publicly report online a mean healing rate of 92%, it is likely that in the real world, among complicated patients, healing rates better than 40.0% are not achievable.
• Criteria needed to report honest healing rates include standardized definitions of “healed wound” and “healing rate,” defined wound care protocols, the inclusion of patient and wound demographics, and the need to report whether any risk stratification was used, if any patients/wounds were not included in the wound healing rate denominator, at what point the healing rate is along the entire episode of care, and the total number of patients/wounds, disaggregated by provider and facility.
• Some basic rules for publicly reporting data have been proposed herein, but other parameters are needed, which should be determined by consensus in the wound care community to ensure that transparent and risk-stratified wound outcome data are reported.

## About the Authors

**Caroline E. Fife, MD,** did her residency in Family Medicine at the University of Texas, Southwestern in Dallas, followed by a Fellowship in Undersea and Hyperbaric Medicine (UHM) at Duke University, where she was subspecialty board–certified in UHM. She was Professor of Medicine in the Division of Cardiology at the University of Texas Health Science Center in Houston (1990–2013). A Certified Wound Specialist since 1998, she is now the Medical Director of the St. Luke's Wound Care Clinic in The Woodlands, Texas, and a Professor of Geriatrics at Baylor College of Medicine in Houston. Dr. Fife is also the Chief Medical Officer of Intellicure, a Texas-based software company, and is the Executive Director of the USWR, a nonprofit organization recognized by CMS as a QCDR. **Kristen A. Eckert, MPhil,** is a certified Medical Writer and Editor (University of Chicago, 2016) with more than 10 years of experience working in global health programs and has contributed to dozens of peer-reviewed medical publications in wound care and other pathologies. **Marissa J. Carter, PhD, MA,** has an MA in biochemistry from Oxford University and a PhD in chemistry from Brandeis University. She is an evidence-based medicine reviewer, biostatistician, and epidemiologist and is President of Strategic Solutions, a company that designs, runs, and analyzes clinical trials, most commonly in wound care.

## Abbreviations and Acronyms

CMS: The Centers for Medicare and Medicaid Services
DFU: diabetic foot ulcer
FDA: Food and Drug Administration
MIPS: Merit-based Incentive Payment System
PQRS: Physician Quality Reporting System
PU: pressure ulcer
QCDR: Qualified Clinical Data Registry
RCT: randomized controlled trial
SD: standard deviation
UHM: Undersea and Hyperbaric Medicine
USWR: U.S. Wound Registry
VLU: venous leg ulcer
